# Memory-inspired spiking hyperdimensional network for robust online learning

**DOI:** 10.1038/s41598-022-11073-3

**Published:** 2022-05-10

**Authors:** Zhuowen Zou, Haleh Alimohamadi, Ali Zakeri, Farhad Imani, Yeseong Kim, M. Hassan Najafi, Mohsen Imani

**Affiliations:** 1 University of California San Diego, La Jolla, CA 92093 USA; 2 University of California Los Angeles, Los Angeles, CA 90095 USA; 3 University of Connecticut, Storrs, CT 06269 USA; 4 Daegu Gyeongbuk Institute of Science and Technology, Daegu, South Korea; 5 University of Louisiana, Lafayette, LA 70504 USA; 6 University of California Irvine, Irvine, CA 92697 USA

**Keywords:** Information theory and computation, Electrical and electronic engineering

## Abstract

Recently, brain-inspired computing models have shown great potential to outperform today’s deep learning solutions in terms of robustness and energy efficiency. Particularly, Spiking Neural Networks (SNNs) and HyperDimensional Computing (HDC) have shown promising results in enabling efficient and robust cognitive learning. Despite the success, these two brain-inspired models have different strengths. While SNN mimics the physical properties of the human brain, HDC models the brain on a more abstract and functional level. Their design philosophies demonstrate complementary patterns that motivate their combination. With the help of the classical psychological model on memory, we propose SpikeHD, the first framework that fundamentally combines Spiking neural network and hyperdimensional computing. SpikeHD generates a scalable and strong cognitive learning system that better mimics brain functionality. SpikeHD exploits spiking neural networks to extract low-level features by preserving the spatial and temporal correlation of raw event-based spike data. Then, it utilizes HDC to operate over SNN output by mapping the signal into high-dimensional space, learning the abstract information, and classifying the data. Our extensive evaluation on a set of benchmark classification problems shows that SpikeHD provides the following benefit compared to SNN architecture: (1) significantly enhance learning capability by exploiting two-stage information processing, (2) enables substantial robustness to noise and failure, and (3) reduces the network size and required parameters to learn complex information.

## Introduction

Many applications run machine learning algorithms to assimilate the data collected in the swarm of devices on the Internet of Things (IoT). Sending all the data to the cloud for processing is not scalable, cannot guarantee a real-time response. However, the high computational complexity and memory requirement of existing DNNs hinder usability to a wide variety of real-life embedded applications where the device resources and power budget is limited^1–4^. Therefore, we need alternative learning methods to train on the less-powerful IoT devices while ensuring robustness and generalization.

System efficiency comes from sensing and data processing. Unlike classical vision systems, neuromorphic systems try to efficiently capture a notion of seeing motion^5–9^. Bio-inspired learning methods, i.e., spiking neural networks (SNNs), address issues related to energy efficiency^5,10–23^. SNNs have been widely used in many areas of learning and signal processing^24–27^. These systems have yet to provide robustness and intelligence that matches that from embodied human cognition. For example, the existing bio-inspired method cannot integrate sensory perceptions with actions. SNN applications in machine learning have largely been limited to very shallow neural network architectures for simple problems. Using deep SNN architecture often does not improve learning accuracy and can result in a possible training divergence^9^. In addition, SNNs lack brain-like robustness and cognitive support.

On the other hand, Hyperdimensional Computing (HDC) is introduced as a promising brain-inspired solution for robust and efficient learning^28^. HDC is motivated by the understanding that the human brain operates on *high-dimensional* representations of data originated from the large size of brain circuits^29^. It thereby models the human memory using points of a high-dimensional space, that is, with *hypervectors*. HDC performs a learning task after mapping data into high-dimensional space. This encoding is performed using a set of pre-generated *base vectors*. HDC is well suited to address several learning tasks in IoT systems as: (i) HDC is computationally efficient and amenable to hardware level optimization^30–32^, (ii) it supports single-pass training with no back-propagation or gradient computation, (iii) HDC offers an intuitive and human-interpretable model^33^, (iv) it is a computational paradigm that can be applied to a wide range of learning and cognitive problems^33–45^, and (v) it provides strong robustness to noise—a key strength for IoT systems^46^. Despite the above-listed advantages, HDC encoding schemes are not designed for handling neuromorphic data. HDC lacks the behavioral resemblance to neurons to extract features from neuromorphic data effectively.

While SNN mimics the physical properties of the brain (how biological neurons are operating), HDC models the brain at a more abstract and functional level. This makes these two computational models complementary. Inspired by the classical and popular memory model, introduced by Atkinson–Shiffrin^47^, we propose a novel framework that fundamentally combines Spiking neural network and hyperdimensional computing. Our framework, called ${\mathsf {SpikeHD}}$, enables a scalable and strong cognitive learning system to better mimic brain functionality. ${\mathsf {SpikeHD}}$ creates a cross-layer brain-inspired system that captures information of sensory data from different perspectives: low-level neural activity and pattern-based neural representation. Since both SNN and HDC have memorization capability, they are powerful in preserving spatial and temporal information. Therefore, ${\mathsf {SpikeHD}}$ can ensure advanced learning capability with high accuracy.

- To the best of our knowledge, ${\mathsf {SpikeHD}}$ is the first framework that fundamentally combines SNN and HDC. ${\mathsf {SpikeHD}}$ first exploits a few layers of spiking neural network to extract low-level spatiotemporal information of raw event-based data. Then, it utilizes HDC to operate over SNN output, learn the abstract information, and classifying the data. To ensure robust, efficient, and accurate HDC learning, we present a non-linear neural encoder that transforms data into knowledge at a very low cost and with comparable accuracy to state-of-the-art methods for diverse applications.
- We develop an end-to-end framework that enables co-training of SNN and HDC models. Instead of using deep SNN architecture, we exploit a simple SNN architecture that updates based on gradient rule and connects it to an HDC module capable of fast and single-pass learning. Our framework trains SNN and HDC models simultaneously to ensure that the data generated by SNN is optimal for HDC learning.
- ${\mathsf {SpikeHD}}$ supports online learning from the data stream. In this configuration, we keep the SNN layer static while exploiting HDC single-pass training capability to update the model in real-time. This enables ${\mathsf {SpikeHD}}$ model to learn or update its functionality with very few samples and without paying the cost of storing large-scale train data for iterative learning.

We evaluate ${\mathsf {SpikeHD}}$ on multiple classification problems. Our evaluation shows that ${\mathsf {SpikeHD}}$ provides significant benefits compared to both HDC and SNN architectures: (1) enhance learning capability by exploiting two-stage information processing, and (2) significantly reduces the network size and required parameters to learn complex information. For example, our results indicate that ${\mathsf {SpikeHD}}$ can provide 6.1% and 3.8% higher classification accuracy on MNIST and DVS Gesture datasets.

## Brain-inspired computing models

The human brain remains the most sophisticated processing component that has ever existed. The ever-growing research in biological vision, cognitive psychology, and neuroscience has given rise to many concepts that have led to prolific advancement in artificial intelligent accomplishing cognitive tasks^41,48,49^.

### Analogy from the brain

In this work, we enhance the machine learning method by exploring and translating the memory processing capability of the brain^47^. To maximize the synergy between anthropogenic concepts and a body *in silico*, we analyzed the distinct neuromorphic nature of SNN and Vector Symbolic Architecture (VSA)^50,51^. We found that the two studies approach neuromorphic computing from complementary philosophies: SNN embodies the sensory processing patterns of the brain from a biological standpoint, while the VSA approach processes data from the behavioral patterns. Finally, we evaluate prototypes in both fields using DECOLLE^52^ (as SNN representative) and Hyperdimensional Computing^28^ (as VSA representative).

#### Memory model

The wildly accepted memory model of Atkinson and Shiffrin^53^ includes sensory registers, short-term store, and long-term store. In particular, short-term store (STS) consists of the memory in storage for a short amount of time (referred to as “short-term memory”) that can be actively engaged in processing (“working memory”). Long-term store (LTS) refers to the memory maintained for long periods of time. Structures centered around the hippocampus serves to process and transfer memory between short-term and long-term storage^54,55^.

#### SNN (DECOLLE) as STS

SNN mimics biological neural networks at the neuronal level, where the representation of the information is the collective state of the spiking neurons, including membrane potential and synaptic states. Given neuromorphic data that are either transformed from frame-based counterparts or captured directly by Dynamic Vision Sensors (DVS), SNN has the structural advantage in accomplishing simple cognitive tasks. In particular, the recurrent nature of DECOLLE renders it ideal for serving both as a sensory processing unit and as an STS.

#### VSA (HDC) as LTS

Hyperdimensional Computing (HDC) mimics the brain on a functional and behavioral level. Just like how the hippocampus represents long-term memory for the sake of efficient storage and retrieval, HDC represents information in hypervectors that can be efficiently and robustly stored in hardware, such as FPGA and GPU, when compared to both non-VSA and most VSA representations. Given informative pieces of data (in this case, the sensory data after being processed by SNN), HDC can efficiently extract higher-level concepts through the process of encoding (what the hippocampus does) and memorization (what the long-term storage does).

### Hyperdimensional computing

The brain’s circuits are massive in terms of numbers of neurons and synapses, suggesting that large circuits are fundamental to the brain’s computing. HDC^28^ explores this idea by looking at computing with ultra-wide words—that is, with very high-dimensional vectors, or hypervectors. The fundamental units of computation in HDC are high dimensional representations of data known as “hypervectors”, which are constructed from raw signals using an encoding procedure. There exist a huge number of different, nearly orthogonal hypervectors with the dimensionality in the thousands^56^. This lets us combine such hypervectors into a new hypervector using well-defined vector space operations while keeping the information of the two with high probability. Hypervectors are holographic and (pseudo)random with i.i.d. components. A hypervector contains all the information combined and spread across all its components in a full holistic representation so that no element is more responsible for storing any piece of information than another.

In recent years, HDC has been employed in a range of applications, such as classification^57^, activity recognition^58^, biomedical signal processing^59^, multimodal sensor fusion^60^, security^61,62^ and distributed sensors^63,64^. A key HDC advantage is its training capability in one or few shots, where object categories are learned from few examples instead of many iterations. HDC has achieved comparable to higher accuracy compared to support vector machines (SVMs)^65,66^, gradient boosting^67^, and convolutional neural networks (CNNs)^33^, as well as lower execution energy on embedded processors compared to SVMs^68^, CNNs and long short-term memory^66^.

### Spiking neural network

Spiking neural networks (SNNs) are brain-inspired solutions for fault-tolerant and energy-efficient signal processing. SNNs take inspiration from the biological functionality of neurons in the human brain to engineering more efficient computing architectures. In the area of machine learning, SNN shares common properties to Recurrent Neural Network (RNNs), such as similarity in general architecture, temporal dynamics, and learning through weight adjustments^69^. Several works are now establishing formal equivalences between RNNs and networks of spiking leaky integrate-and-fire (LIF) neurons which are widely used in computational neuroscience^70^. In the LIF model, the neuron’s state is the momentary activation level that can be pushed higher or lower depending on the incoming spike value. The neuron state will be reset to a lower value after firing the state^71^.

The complicated dynamics of the biological spiking neuron has posed great difficulty in designing efficient SNNs capable of solving complex information processing problems^72^. Early models has relied on Spike Time Dependent Plasticity (STDP) that depends on pre-synaptic and post-synaptic information^73,74^, which results in non-differentiable models^73,74^. Surrogate gradients and multiple learning schemes has been proposed to tackle this challenge. For example, spatio-temporal backpropagation (STBP)^16^ uses an approximated derivative for spike activity that combines spatial and temporal domain to allow gradient descent^75^ proposed Spike-timing-dependent back-propagation (STDBP) enabled by Rectified linear postsynaptic potential function (ReL-PSP) to learn in an event-driven manner. Aggregate-label learning such as efficient threshold-driven plasticity (ETDP) algorithm also demonstrated ability to learn useful multi-modal sensory clues efficiently^76^. Finally, progressive tandem learning of SNNs^77^ and VGG and residual architectures^9^, which applies an ANN-to-SNN conversion and layer-wise learning framework, has also showed efficient learning capabilities in classification and regression tasks. In addition to the surrogate gradient methods that constitute a large portion of the literature, many neuron models that compute exact gradients has also been proposed, such as EventProp^78^, first-spike-time learning^79^, and Temporal Coding with Alpha Synaptic Function^80^. Biologically plausible models such as E-prop^81^ have also been of interests, and readers are referred to^82^ for an in-depth survey.

Other significant results on biologically plausible learning with SNN, such as E-Prop^9^ are also not mentioned, and are directly comparable to DECOLLE.

Several existing hardware solutions have focused on their implementation in the forms of unsupervised and semi-supervised learning^83,84^. However, these works are limited in learning static patterns or shallow networks. Recent breakthrough research shows that Deep Continuous Local Learning (DECOLLE) provides effective and efficient training with approximate loss that maintains synaptic plasticity. Unlike conventional surrogate gradient learning, the cost function of DECOLLE is local in time and space, such that only one trace per input neuron is required. This enables the algorithm to scale linearly in space. Furthermore, in DECOLLE the computation of the gradients can reuse the variables computed for the forward dynamics, so learning has no additional memory overhead^24,25^.

## ${\mathsf {SpikeHD}}$ overview

In this paper, we propose ${\mathsf {SpikeHD}}$, the first hybrid solution that fundamentally combines spiking neural networks and hyperdimensional computing. Our framework exploits SNN and HDC in the following ways:

- *Spiking neural network* SNN extracts low-level information of neuromorphic data. SNN is like a feature extractor that learns spatiotemporal information of noisy spikes and projects them into meaningful representation. SNN eliminates noisy events that are less frequent in a temporal manner and exploits redundancy to further strengthen spatially correlated information. DECOLLE, in particular, uses deep continuous local learning, where the network errors are computed within each layer, thus requiring little memory overhead for computing gradients.
- *Hyperdimensional encoding* HDC performs a higher level learning over spike data generated by SNN. As explained in “Introduction”, HDC consists of two layers: encoder and learner. The encoder maps SNN output spikes into high-dimensional space. HDC encoder is unsupervised and significantly efficient since it does not require any training process. Since the encoder is non-linear, a single-layer HDC classifier can effectively learn the data. The training only operates over the HDC model to keep efficiency and does not propagate to the encoder module.

**Figure 1:**
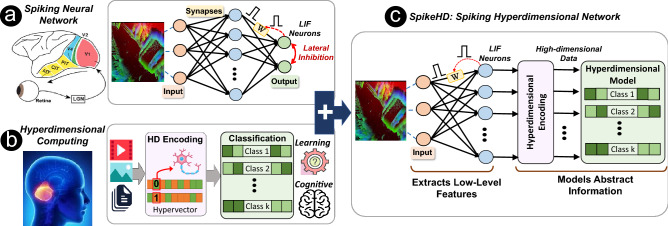
${\mathsf {SpikeHD}}$ overview architecture: (**a**) spiking hyperdimensional neural network (Reprinted with permission from reference^85^. Copyright 2012 Elsevier); The leaky-integrate-and-fire (LIF) layers of the SNN have a high synaptic resemblance to the neuronal systems in the brain. This gives rise to its advantage in (the learning of) sensory data processing and maintaining working memory for classification. (**b**) The high-dimensionality and holography of HDC renders a cerebellum-like functionality with a high capacity memory. (**c**) the combination of the two as guided by the Atkinson-Shiffrin memory model allows ${\mathsf {SpikeHD}}$ to take advantage of both and overcome their respective shortcomings.

### How to combine SNN and HDC

A naive approach can use SNN and HDC in parallel or in series to make a prediction. In the parallel version, both SNN and HDC can make independent predictions, and we can make decisions by looking at the decisions along with their confidence. However, this approach has the following challenges: (1) high computational cost to train two separated models, and (2) decision making and trust in the model is a complex task and requires another learning model. Similarly, the serial connection of SNN and HDC has the following challenges: (1) an information flow that is limited between the models impedes the ability of both: the latter fails to make good predictions due to a lack of information, and the former fails to be updated due to a lack of loss inferred from the prediction. (2) SNN and HDC are working over different data representations and update rules. SNN works with spike data and is trained using gradient-based rule, while HDC works using dense high-dimensional data representation. This makes HDC and SNN learning not compatible and their interactions non-trivial.

### Our contribution

To get simultaneous benefits from SNN and HDC, ${\mathsf {SpikeHD}}$ needs to combine them based on their strengths and capabilities. Figure 1 shows an overview of our hybrid ${\mathsf {SpikeHD}}$ operating over neuromorphic data. ${\mathsf {SpikeHD}}$ exploits two layers of information extraction from event-based sensors: (1) SNN layer to extract low-level information by preserving spatiotemporal correlation of data and (2) hyperdimensional computing to learn the abstract and high-level trend of data. ${\mathsf {SpikeHD}}$ developed a novel framework that co-trains SNN and HDC models. The co-training enables the interaction between SNN and HDC to ensure convergence towards an optimal model. As Fig. 1 shows, hyperdimensional learning, which operates over the spike data, has two components: a non-linear neural encoder that maps SNN output to high-dimensional space and an HDC learning model that combines encoded hypervectors to generate a hypervector representing each class. The HDC model will always take the final prediction.

In “Introduction”, we explained the details of HDC learning. In the rest of the proposal, we present our framework that integrates SNN and HDC (“[Sec Sec12]”).

## ${\mathsf {SpikeHD}}$: integration of brain models

**Figure 2:**
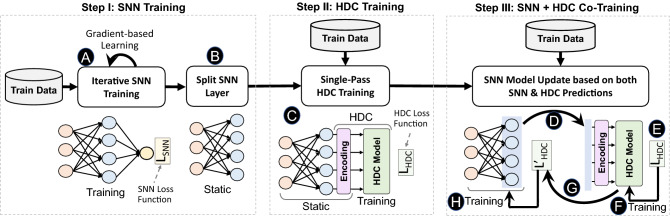
SpikeHD training process: *Step I: SNN iterative training.* An original instance of SNN is trained without the influence of HDC, in which initial feature extraction recurrent learning from neuromorphic data are established. *Step II: HDC single-pass training.* The HDC module is first injected into a deep SNN layer, and train data is propagated from the SNN input layer forward through the HDC module to make a prediction, which leads to modification in the HDC memory component. *Step III: SNN and HDC co-training.* the loss from the output of HDC module is used to update both HDC memory and SNN layers simultaneously.

In this section, we propose a novel framework to combine spiking neural networks and hyperdimensional computing. Figure 2 shows an overview of our framework combining SNN and HDC.

### Step I: SNN training

Our first step aims to establish feature extraction and to fine-tune the short-term memory behavior of the model. The solution starts by training the original SNN model, implemented with DECOLLE, using an entire or a batch of train data. The SNN is a multi-layer network that gets spike data as an input and makes a learning decision on the output layer. Depending on the loss function defined on SNN output, the SNN uses a gradient-based rule to update the synapse’s weights (A). During this phase of the training, the SNN learns to extract information from noisy neuromorphic data. Because the synaptic plasticity rules are partially derived from the neural dynamics of the spiking neurons, it is capable of learning spatiotemporal patterns. Increasingly complex features can be learned by adding layers to the SNN, but it is costly and introduces difficulty in convergence. Thus, the number of layers is often limited.

### Step II: HDC training

After training the SNN to approximate convergence, the solution applies *HDC injection*: we split SNN from early layers (often a relatively deep layer) and connect it to the HDC module to extract more abstract information (B). In this step, the SNN layers are considered to be static because no training happens on SNN. For each train data, we first pass data through SNN. Then, we will be given the SNN representation in the split layer as labeled data to the HDC module for training. HDC encoding serves as the “hippocampus” and maps the spike data into high-dimensional space. Due to the non-linearity of the encoder, an efficient HDC learning module can effectively learn the pattern of data (C). One advantage of HDC is its capability in learning a one-pass classification model. This eliminates the necessity of a costly iterative method to train a model. As the learning heavily depends on memorization of the encoded hypervectors, the HDC model serves as the long-term memory of the model.

### Step III: SNN and HDC co-training

The current approach trains SNN and HDC sequentially, where the SNN training does not get any feedback from HDC module. The SNN module is trained based on the loss function defined at the SNN output layer. However, as we explained, our hybrid architecture performs the prediction using the HDC model. This results in sub-optimal training of ${\mathsf {SpikeHD}}$ architecture.

To address this issue, we propose a novel co-training method that enables SNN to be trained based on the HDC model prediction. To ensure the SNN layer is well trained for HDC prediction, ${\mathsf {SpikeHD}}$ retrains the SNN layers after HDC training. For every training data or batch of data, ${\mathsf {SpikeHD}}$ starts updating the SNN as follows: Firstly, the spike data passes through the split SNN layer (D) up until the point of injection and generates vectors as input to the HDC module. Next, the HDC module encodes the input, which is then compared to the HDC model; the loss function is computed against the target label (E). After that, the loss is used to update the HDC model in a single-pass. The learning in HDC is pattern-based and can perform with significantly higher computation efficiency (F). Then, HDC back-propagates the loss through the HDC module back to the point of injection (G). Finally, we update the SNN model using a gradient-based rule with the backpropagated loss (H). This procedure continues iteratively over train data until finding a suitable SNN representation that can ensure maximum prediction accuracy in HDC output.

One thing to note is that the back-propagation in (F) mainly concerns two matrix multiplications with no significant overhead during training. Passing through the HDC model requires multiplying the loss with the HDC model itself, which generates a loss hypervector. To pass the loss hypervector through the static encoder, we apply the inverse of the activation function and an inverse of the encoding matrix. Since the encoding matrix is not invertible, the Moore–Penrose pseudoinverse of the matrix is applied, and it can be pre-computed upon the initialization of the model. Since the HDC model has faster training than SNN, one can decide to update the HDC model less frequently during the co-training step. During co-training, the HDC loss function can be back-propagated and used to update the SNN layer while the HDC model stays constant. The HDC model update can happen less frequently to ensure lower training costs.

### SpikeHD online learning

Despite the effectiveness of the proposed framework, the training hybrid SNN and HDC model can be costly for small embedded devices. Our framework requires iteratively repeat co-training phases, which often require a large number of data and iterations to update the quality of the SNN model. Embedded devices may have the following limitations to implement ${\mathsf {SpikeHD}}$ iterative learning: (1) embedded devices often do not have enough memory to keep all train data for iterative learning. As a result, to enable online learning, ${\mathsf {SpikeHD}}$ needs to be updated in one-pass with no need to store train data. (2) embedded devices have limited resources which may not be enough to support the costly gradient-based model update required by SNN. Here, we propose a solution that enables online ${\mathsf {SpikeHD}}$ learning. As explained in “Introduction”, HDC supports single-pass training by creating a model with one-time looking at train data. We exploit this feature to update ${\mathsf {SpikeHD}}$ model in real-time based on the data stream. In this configuration, the majority of our learning relies Step II, with limited training on Step I and no required training on step III. In particular, the SNN is first trained with very few data points to learn feature extraction. Then, the SNN model is assumed to be static and does not get updated. Instead, for each batch of data, ${\mathsf {SpikeHD}}$ only updates the HDC model based on the generated loss function. This is similar to transfer learning, where the SNN knowledge is used for new data or environments. This model results in much faster convergence and eliminates the necessity of storing train data.

### ${\mathsf {SpikeHD}}$ scalability & robustness

${\mathsf {SpikeHD}}$ hybrid architecture provides an advanced brain-inspired learning solution with multi-layer information processing. This architecture is significantly strong in preserving spatiotemporal information. ${\mathsf {SpikeHD}}$ also provides the following advantages:

- *Scalability* Using deep SNN architecture often does not improve learning accuracy or results in a possible divergence. ${\mathsf {SpikeHD}}$ hybrid architecture enables SNNs to use effective shallow networks rather than deep non-scalable networks. HDC encoding is used as secondary information processing to provide a high quality of learning while ensuring fast and scalable SNN training.
- *Robustness* HDC encoding is holographic and redundant, thus provides significant robustness to noise and failure. Our represents stores information of events as a pattern of neural activity in high-dimensional space. Therefore, losing a single or series of dimensions would not remove the information of an event. We further explore on ${\mathsf {SpikeHD}}$ robustness in “[Sec Sec23]”.

## Evaluation

We evaluate the classification performance of ${\mathsf {SpikeHD}}$ on two benchmarks: DVS Gesture Dataset^86^ and spike-trained MNIST^87^. DVS Gesture Dataset is obtained by Dynamic Vision Sensor (DVS) capturing 11 types of hand and arm gestures from 29 distinct subjects under 3 different lighting conditions, and we downsized the event streams from $128 \times 128$ to $32 \times 32$ and binned in frames of 1*ms* for efficient tra^52^. It is thus natively neuromorphic. Spike-trained MNIST, in contrast, is artificial. It comes from processing the original frame-based MNIST images to spike trains, where the serialized pixel values determine the firing rate of the simulated sensors. We used the poisson model of spike generation with 1000 timesteps for converting spike-trained MNIST, which was available as part of DECOLLE codebase^52^.

The proposed ${\mathsf {SpikeHD}}$ framework has been implemented with two co-designed modules: spiking neural network and hyperdimensional computing. For SNN, we use the existing open-source library^52^ that trains a network using DECOLLE. For HDC, we have developed an in-house library compatible with PyTorch. Our library is an optimized version of PyTorch that better handles the HDC memory requirement for CPU and GPU. The default parameters of ${\mathsf {SpikeHD}}$ is as follows. The SNN component consists of 5 LIF layers with respectively 150, 120, 100, 120, and 150 neurons. Each LIF layer is associated with a readout layer and a dropout layer as made necessary by local learning. Time constants that capture the decay dynamics of spiking neurons are $\alpha = 0.9$ and $\beta = 0.85$. For the HDC component, a dimension of $D=4000$ is used, and the HDC encoder utilizes the hyperbolic tangent function (Tanh) as the activation function. The injection depth that indicates the layer of SNN where HDC is injected is by default 4, which means that it is injected right before the last LIF layer. During training, we used a smooth L1 loss function for Step I, similar to^52^, and L1 loss function for Step II and III. We used AdaMax Optimizer with parameters $\beta _1 = 0$, $\beta _2 = 0.95$, and $lr = 10^{-9}$. Finally, the default dataset for evaluation is spike-trained MNIST.

### Quality of learning

**Figure 3:**
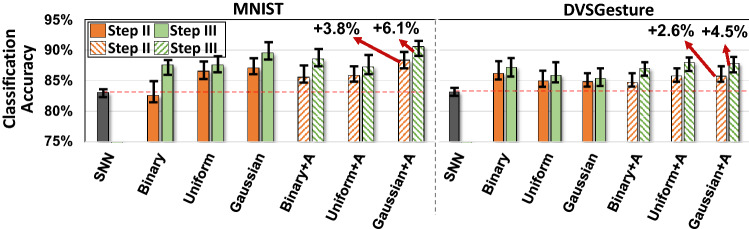
Training performance of ${\mathsf {SpikeHD}}$ with distinct HDC encoders on MNIST and DVSGesture. Each bar plot represent the accuracy of SNN along with ${\mathsf {SpikeHD}}$ using HDC encoders with binary, uniform, or Gaussian base hypervectors. For HDC encoder, the results are reported with and without an activation function (+A indicates an encoder with *Tanh* activation.). The error bars are shown when repeating each experiment for 20 times.

Figure 3 compares the test classification accuracy of ${\mathsf {SpikeHD}}$ with DECOLLE. For SNN, we use the fully connected DECOLLE architecture in our default configuration. For HDC, we adopt HDC models to directly encode and learn from neuromorphic data with the default dimension and HDC encoder. For different instances of ${\mathsf {SpikeHD}}$, we apply the default parameters except for our variable—HDC encoder. The models are trained iteratively for 40 epochs, all of which reached convergence.

Our evaluation shows that default ${\mathsf {SpikeHD}}$ outperforms both SNN and HDC in terms of quality of learning. HDC model alone provides the lowest classification accuracy, as the HDC encoder is weak in extracting spatial and temporal information from noisy spike data. In other words, HDC learning is abstract and cannot be well adapted to extract low-level information from neuromorphic data. In contrast, SNN naturally models the brain’s visual systems, thus providing high classification accuracy. However, the SNN accuracy saturates with the increase in the number of layers. In contrast, ${\mathsf {SpikeHD}}$ is a powerful classifier that extracts multi-layer information from the neuromorphic data. Therefore, it eliminates the necessity of using deep SNN layers. Our evaluation shows that ${\mathsf {SpikeHD}}$ achieves, on average, 5.7% and 3.2% higher classification accuracy compared to the SNN model after co-training on MNIST and DVS Gesture, respectively.

Table 1 compares the test classification of convolutional ${\mathsf {SpikeHD}}$ with state-of-the-art SNNs. For network architecture with only fully connected layers, the comparisons are less insightful, as we were not able to find classification results for deep spiking neural networks on MNIST. The performance of shallow and fully connected DECOLLE is similar to that of the deep one demonstrated in Fig. 3, but ${\mathsf {SpikeHD}}$ does not improve upon its accuracy as expected. While the test accuracy of both DECOLLE and ${\mathsf {SpikeHD}}$ in the fully connected setting are lower than those of EventProp and STDBP, convolutional ${\mathsf {SpikeHD}}$ is able to improve upon its base DECOLLE and achieve comparable accuracy to STDBP. This implies the opportunity for ${\mathsf {SpikeHD}}$ to improve upon other models through the combination illustrated in “[Sec Sec12]”.

### Hyperdimensional encoding

To show the impact of our HDC encoder, Fig. 3 also compares ${\mathsf {SpikeHD}}$ accuracy when HDC is using different encoding modules: linear (Binary)^33,89^, random projection (Uniform)^66,90^, and our proposed non-linear encoder (Gaussian). Our evaluation shows that ${\mathsf {SpikeHD}}$ using a non-linear encoder provides significantly higher classification accuracy compared to the other encoders, independent of the base hypervectors. In particular, linear encoding (labeled as ‘Binary’ in Fig. 3) provides the lowest accuracy among them due to limited HDC memory capacity—4000 bits per class—compared to the ones with non-binary base vectors. The higher accuracy of our encoding comes from: (1) ${\mathsf {SpikeHD}}$ capability in considering the interactions between the features, and (2) exploiting an activation function that makes the mapping non-linear. Our evaluation shows that ${\mathsf {SpikeHD}}$ utilizing non-linear encoder achieves, on average, 3.1% and 2.4% higher quality of learning compared to linear and random projection encoder.

Our evaluation also showed that there is progressive improvement in the learning accuracy as the model proceeds with training steps. In most cases, the test accuracy has significant improvement from step I to step II, and slightly less from step III. We will discuss the possible causes in the later evaluations.

**Table 1:** Comparison of published classification test accuracy on MNIST for SNNs and State-of-the-art ANN and their respective architectures. The naming of network architectures follows^75^, where layers are separated by—and spatial dimensions are separated by ×. The convolution layer and pooling layer are represented by C and P, and the hyperdimension memory, unique to ${\mathsf {SpikeHD}}$, is represented by H.

Model and algorithms	Network architecture	Accuracy (%)
DECOLLE	16$\times$16C7-P2-32$\times$32C7-P2-64	98.0
EventProp^78^	784-350-10	97.6
STDBP^75^	28$\times$28-16C5-P2-32C5-P2-800-128-10	99.4
HVCs^88^	*State-of-the-art ANN model	99.8
SpikeHD	16$\times$16C7-P2-32$\times$32C7-P2-64-4kH	99.2

### ${\mathsf {SpikeHD}}$ training phases

**Figure 4:**
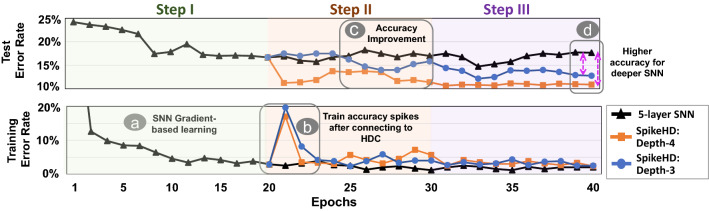
Average train and test accuracy of ${\mathsf {SpikeHD}}$ models with a 5-layer DECOLLE and HDC injection at various depths, compared with pure DECOLLE trained for the same number of epochs. Step 1, training SNN only, consists of 20 epochs and reaches model convergence; step 2, training HD module only, and step 3, co-training, both consist of 10 epochs, as convergence is reached earlier in general.

Figure 4 shows the effect of depth of HDC injection on ${\mathsf {SpikeHD}}$ classification accuracy during both train and test phases. The results are reported for ${\mathsf {SpikeHD}}$ in comparison with DECOLLE with our default setting.

During step I of the training (Fig. 4a), the accuracy of both the training and testing data quickly increases and stabilizes. This implies the efficiency and effectiveness of DECOLLE in simulating and processing spike data, despite its limited generalization as indicated by the non-trivial difference between test accuracy and train accuracy.

When the model enters step II, the training accuracy experience a sharp drop at epoch 20 because we switch from the latter LIF layer to HDC module (Fig. 4b). The cause is obvious: since the newly introduced HDC memory is initially a random model, it has no predictive power. That said, the train/test is then reduced and stabilized in about one epoch. In addition, test accuracy experience an improvement (Fig. 4c) for the displayed depths of injection, which indicates that HDC modules further extracted and memorized features from the DECOLLE layers that are useful to the prediction. We omitted the result of the trivial model with HDC-injection at depth-5 because it achieves similar performance as the baseline (pure SNN). This is because the depth-5 HDC module connects to the last layer of SNN, which essentially takes the prediction produced by SNN and does learning based on that.

Finally, the model enters step 3 at epoch 30. Notice that the training accuracy does not have any improvement while the test accuracy has its final improvement (Fig. 4d) most accentuated for the depth-3 model. This indicates that the improvement in test prediction comes from the update to the SNN layer in conjunction with that to the HDC memory.

As results indicate, the original SNN network suffers from overfitting, as demonstrated by the difference in train and test accuracy due to the depth of the network. In comparison, ${\mathsf {SpikeHD}}$ mitigates this issue by introducing more explicit memory based on non-linear encoding and is less sensitive to the number of layers. Starting from depth-4, ${\mathsf {SpikeHD}}$ observes significant improvement on the prediction, and the best performance is at depth-4 with the parameters we have chosen; this is partly due to DECOLLE’s capacity to extract more meaningful features in the deeper layers, and the long-term memory that the HDC model provides gives rise to increased testing accuracy and, if not eliminated, mitigated the overfitting of DECOLLE model (Fig. 4d).

### ${\mathsf {SpikeHD}}$ online learning

**Figure 5:**
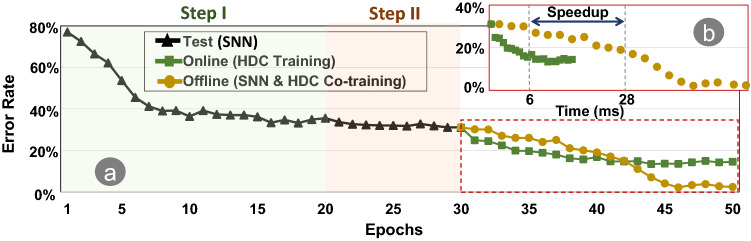
Online and offline training performance by epoch and by running time. (**a**) demonstrates training progress of step I and II with limited training data (100 samples for MNIST). Then, it is trained with the rest of the data either online, where only the HDC part is updated, or offline, where the co-training happens. (**b**) demonstrates the training progress measured by running time, with step II and online/offline step, the former of which is scaled according to training data size for reference.

Figure 5 compares ${\mathsf {SpikeHD}}$ training efficiency during ${\mathsf {SpikeHD}}$ offline and online training. For fairness, both methods perform Step I and Step II ${\mathsf {SpikeHD}}$ training over a small portion of train data. For the rest of the train data, ${\mathsf {SpikeHD}}$-offline continues updating the model by co-training SNN and HDC (Step III defined in our framework), while ${\mathsf {SpikeHD}}$-online only updates the HDC. In other words, ${\mathsf {SpikeHD}}$-online keeps SNN layers fixed and transfers the learned knowledge for the rest of train data.

Notice firstly that even with only 100 MNIST samples, 10 for each class, DECOLLE was able to extract many meaningful features. This is implicated by the immediate increase in test accuracy in Step I (see Fig. 5). In Step II, the epoch-wise and time-wise convergence results are reported for both offline and online methods. The time-wise graph is shown as one epoch has different times in offline and online techniques. Our results indicate that the online method reaches convergence quicker than the offline method, though the offline method may perform better upon convergence. We observe that the training time of the offline method is much longer than the online learning method. This is partly due to DECOLLE’s local training. We see from the convergence speed and the accuracy improvement that (1) online training incorporates new samples quickly into HDC memory, and (2) the co-training succeeds in backpropagating the loss to the SNN module so that it gets updated for better performance.

Our evaluation shows that ${\mathsf {SpikeHD}}$-online can provide comparable accuracy to ${\mathsf {SpikeHD}}$-offline learning method even when the initial training is very limited (100 samples). In other words, ${\mathsf {SpikeHD}}$ can ignore costly iterative training for a big portion of train data. Instead, it simply updates the HDC model at a minimal cost. Our evaluation shows that ${\mathsf {SpikeHD}}$-online can significantly speedup the training process and also reduces the memory footprint required for training. For example, ${\mathsf {SpikeHD}}$-online enables 4.6 × faster and 3.1 × lower trainable memory while ensuring the same quality as an online model.

### ${\mathsf {SpikeHD}}$ accuracy and robustness vs. HDC dimensionality

**Figure 6:**
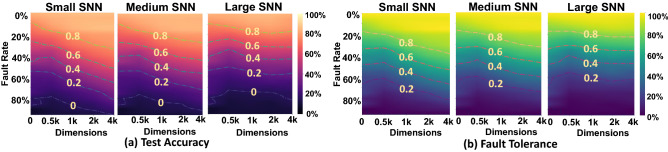
The test accuracy and fault tolerance of ${\mathsf {SpikeHD}}$ under different SNN and HDC parameter settings. (**a**) Test accuracy is measured by the classification accuracy of the MNIST test data from the model given SNN size, dimension of the HDC memory, and proportion of faulty parameters. (**b**) Fault Tolerance is measured by the quality of prediction sustained under memory failure, using its 0-fault counterpart and a random classifier (which has a test accuracy of 0.1 in the case of MNIST) as reference.

Dimensionality creates a trade-off between three ${\mathsf {SpikeHD}}$ parameters: accuracy, efficiency, and robustness. Figure 6 shows the impact of hypervector dimension on ${\mathsf {SpikeHD}}$ test accuracy (considering 0% error rate). ${\mathsf {SpikeHD}}$ in higher dimensionality is a powerful model that can effectively learn the SNN output patterns. ${\mathsf {SpikeHD}}$ provides maximum accuracy even when the dimension reduces from $D=8k$ to $D=4k$. Further decreasing the dimensionality from $D=4k$ results in a minor effect on ${\mathsf {SpikeHD}}$ quality of learning. For example, ${\mathsf {SpikeHD}}$ in $D=2k$ and $D=1k$ provides only 2.3% and 3.5% quality loss compared to ${\mathsf {SpikeHD}}$ model in full dimensionality ($D=8k$). ${\mathsf {SpikeHD}}$ efficiency also directly depends on the model dimensionality. A higher dimensionality increases the number of required computations in both train and test. However, because the accuracy of HDC modules resembles a sigmoid function along the dimension, to reduce the computation cost, one can decide to use ${\mathsf {SpikeHD}}$ in lower dimensionality. For example, reducing ${\mathsf {SpikeHD}}$ dimension from $D=4k$ to $D=2k$ ($D=1k$) results in 1.7 × (3.1 ×) faster computation.

We compare ${\mathsf {SpikeHD}}$ computational robustness with SNN. Our evaluation shows that ${\mathsf {SpikeHD}}$ HDC module significantly improves SNN robustness to possible noise and failure. Figure 6 shows ${\mathsf {SpikeHD}}$ accuracy when losing a different proportion of random neurons in the model. The results are reported for ${\mathsf {SpikeHD}}$ using different dimensionality and using different size SNN networks. Our evaluation shows that ${\mathsf {SpikeHD}}$, in general, provides higher robustness than existing SNN, especially when ${\mathsf {SpikeHD}}$ dimensionality increases. For example, under 10% random noise, ${\mathsf {SpikeHD}}$ and SNN maintain 94.0% and 87.1% quality. The ability to sustain prediction quality generally increases as the dimension of HDC memory increases, though it does generate a slight dip when the HDC dimension is low. This can be attributed to the fault tolerance of DECOLLE and the higher vulnerability of low-dimensional HDC modules. Our results indicate that test accuracy has only a slight advantage when the HDC dimension is high, and SNN is large. This advantage will be more accentuated in smaller SNN, or in more complex tasks.

## Conclusion and discussion

**Figure 7:**
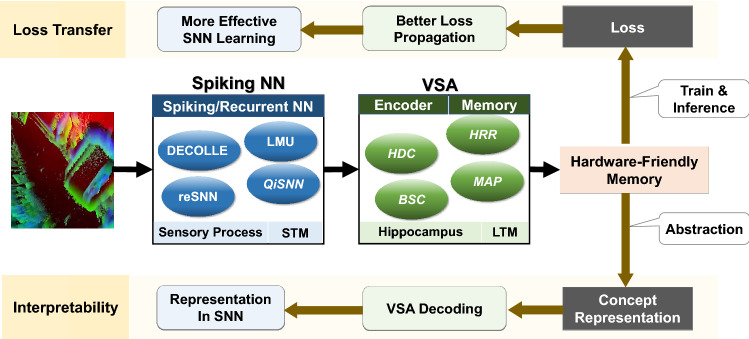
Overview of ${\mathsf {SpikeHD}}$ extended. *Framework* ${\mathsf {SpikeHD}}$ incorporates spiking neural networks and vector symbolic architecture at a high level. While our work has demonstrated one efficiency-oriented instance of the model with DECOLLE and HDC, other combinations may give rise to models of different strengths. *Loss transfer* one direction for future work is the back-propagation of loss from VSA. Better loss propagation to the SNN layer leads to more effective SNN training. *Interpretability* The decoding of HDC Memory and the interpretability of SNN lead to knowledge at the sensory data level.

In this paper, we propose ${\mathsf {SpikeHD}}$, a novel framework that combines Spiking neural network and hyperdimensional computing in order to design a scalable and strong cognitive learning system that better mimics brain functionality. ${\mathsf {SpikeHD}}$ exploits spiking neural networks to extract low-level features by preserving the spatial and temporal correlation of raw event-based spike data. Then, we utilize HDC to operate over SNN output by mapping the signal into high-dimensional space, learning the abstract information, and classifying the data. Our evaluation on a wide range of classification problems shows that ${\mathsf {SpikeHD}}$ provides significant benefit compared to both HDC and SNN architecture: (1) enhances learning capability by exploiting two-stage information processing, (2) significantly reduces the network size and required parameters to learn complex information. For the rest of this section, we highlight some of the open challenges that our framework has yet to overcome and encourage exploration of the question along multiple axes (Fig. 7).

### Loss backpropagation

During step III of ${\mathsf {SpikeHD}}$, a Moore–Penrose inverse of the HDC encoder is applied to backpropagate the loss from the HDC module to the SNN. Since HDC encoder maps vectors to hypervectors, the rank of the inverse is limited to the output dimension of the SNN at the point of injection, which may be way less than the dimension. A large amount of information may be lost from this transition. We experimented with several methods to solve this problem. One example is to continue training the original SNN, transfer the new weights to the ${\mathsf {SpikeHD}}$, and then train the HDC module. This did not improve performance except in the case of the transfer learning task, where the context of the data or the task changes. One method that may be suggested is to introduce a regularization term in the loss function of the SNN layers such that it outputs an HDC-like vector as the representation of the data directly. This will avoid the explicit usage of the HDC non-linear encoder, and the loss will be optimally propagated up to the approximation introduced by the regularization term.

### Component choices

We have selected DECOLLE as SNN and HDC as VSA for our hybrid model for the reason we’ve discussed in section 2.1. It is optimized for time and energy efficiency, and practicality, as both models are known for such traits. Readers interested in the exploration of other aspects may choose to adopt our memory framework to other components. such as Legendre Memory Units^91^ and HDC, or BI-SNN and HRR^92^.

### Concept interpretability

Our current usage of the memory framework is to directly operate on long term memory and derives decisions from its representation, which simulates what the cerebellum does. For the purpose of completing the analogy to the Atkinson–Shiffrin memory model, it has yet to be incorporated the decoding mechanisms of the HDC memory: we did not fetch the long term memory back to the hippocampus and decode it for operations in working memory. This subject is not the purpose of this paper, and the decoupling of encoding and decoding invites more possibilities, as the HDC memory may be used as heterogeneous storage such that multiple tasks may be performed in one memory model.

That said, the subject of interpretability remains interesting at two levels. Each entry in the HDC memory represents the concept of the class, which can be decoded to retrieve a representation of the concept at the SNN level. The representation in SNN can then be interpreted to infer knowledge on the original sensory data.

## Supplementary Information

Supplementary Information.

## Data Availability

The datasets analysed during the current study are DVS Gesture Dataset^86^ and spike-trained MNIST^87^ which are available online for public use.
