# Supplementary material for: Memory-inspired spiking hyperdimensional network for robust online learning
Source: Sci Rep. 2022 May 10;12:7641. doi: 10.1038/s41598-022-11073-3 (PMC9090930; doi:10.1038/s41598-022-11073-3)
Supplement: Supplementary file 1 — Supplementary Information. [file 41598_2022_11073_MOESM1_ESM.pdf]

Supplementary material for "Memory-Inspired Spiking Hyperdimensional Network for Robust Online Learning"

1 Hyperdimensional Classification

We present a robust and lightweight hyperdimensional classification. Figure S1a shows an overview of HDC learning. The first step in HDC is to encode data into high-dimensional space. Then, HDC performs a learning task over encoder data by performing a single-pass training. The result of training will be to generate a hypervector representing each class. The inference task can perform by checking the similarity of an encoded query to the class hypervector.

1.1 Non-Linear Encoding

The encoder is the most important component of hyperdimensional learning. The goal of the encoder is to map data into high-dimensional space such that we can extract knowledge from data at a lower cost. The encoder selection depends on the data type (structure of data) and the target learning task. There are multiple existing HDC-based encoding methods [33, 93, 94]. Although these methods have shown excellent classification accuracy for their application-specific problems, to the best of our knowledge, the existing encoding methods linearly combine the hypervectors corresponding to each feature, resulting in sub-optimal classification quality for general classification problems. To obtain the most informative hypervectors, the HDC encoding should consider the non-linear interactions between the feature values with different weights.

In this context, we propose a novel encoding method that exploits the kernel trick [95, 96] to map data points into the high-dimensional space. The underlying idea of the kernel trick is that data, which is not linearly separable in original dimensions, might be linearly separable in higher dimensions. Let us consider certain functions $K(x, y)$ which are equivalent to the dot product in a different space, such that $K(x, y) = \Phi(x) \cdot \Phi(y)$, where $\Phi(\cdot)$ is often a function for high dimensional projection. The Radial Basis Function or Gaussian Kernel is the most popular kernel:

$$K(x,y) = e^{\frac{-||x-y||^2}{2\sigma^2}}$$

We can take advantage of this implicit mapping by replacing their decision function with a weighted sum of kernels:

$$f(\cdot) = \sum_{i=0}^{N} c_i K(\cdot, x_i)$$

where (x_i, y_i) is the training data sample, and the c_is are constant weights. The study in [95] showed that the inner product can efficiently approximate Radial Basis Function (RBF) kernel, such that:

$$K(x,y) = \Phi(x) \cdot \Phi(y) \approx z(x) \cdot z(y)$$

The Gaussian kernel function can now be approximated by the dot product of two vectors, $z(x)$ and $z(y)$.

The proposed encoding method is inspired by the RBF kernel trick method. Figure S1b shows our encoding procedure. Assume an input vector in original space $\vec{F} = \{f_1,\ f_2, \cdots, f_n\}$ and $F \in \mathcal{R}^n$. The encoding module maps this vector into high-dimensional vector, $H = \{h_1,\ h_2, \cdots, h_D\} \in \mathcal{R}^D$, where $D \gg n$. The following equation shows an encoding method that maps input vector into high-dimensional space:

$$h_i = \cos(\vec{F} \cdot \vec{\mathcal{B}}_i + b_i) \sin(\vec{F} \cdot \vec{\mathcal{B}}_i) \tag{S1}$$

where $\vec{\mathcal{B}}_k$s are randomly chosen hence orthogonal base hypervectors of dimension $D \simeq 10k$ to retain the spatial or temporal location of features in an input and $b_i \sim \mathcal{U}(0, 2\pi)$. That is, $\vec{\mathcal{B}}_{kj} \sim \mathcal{N}(0,1)$ and $\delta(\vec{\mathcal{B}}_{k_1}, \vec{\mathcal{B}}_{k_2}) \simeq 0$, where δ denotes the cosine similarity. However, this activation is not a convex function,

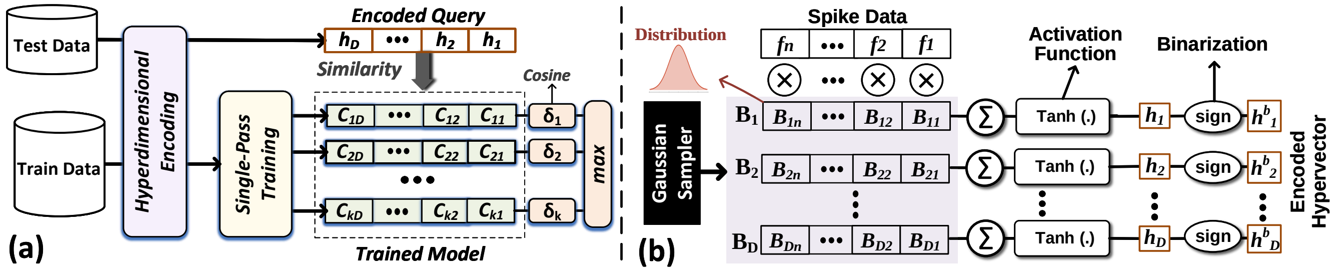

Figure S1: (a) Overview of hyperdimensional classification during both training and testing phases, and (2) HDC non-linear encoder.

thus making it impossible to back-propagate from HDC encoder. Although HDC learning does not rely on back-propagation, in section 4.3, we will show that the integration of SNN and HDC requires back-propagation through HDC encoding module. To address this issue, we exploit hyperbolic tangent function (Tanh) as an activation function:

$$h_i = \tanh(\vec{F} \cdot \vec{\mathcal{B}}_i + b_i) \tag{S2}$$

After this step, each element h_i of a hypervector $\mathbf{H}^n$ has a non-binary value. In the HDC, binary (bipolar) hypervectors are often used for computation efficiency. We thus obtain the final encoded hypervector by binarizing it with a sign function ($\mathbf{H} = sign(\mathbf{H}^n)$) where the sign function assigns all positive hypervector dimensions to '1' and zero/negative dimensions to '-1'. The encoded hypervector stores the information of each original data point with D bits. In our example, the feature vector, F, is highly sparse event-based data. However, after mapping data through the above encoder, the generated high-dimensional data will get a dense binary representation.

1.2 Hyperdimensional Model Training

We exploit hyperdimensional learning to directly operate over encoded data. Figure S1a shows an overview of HDC classification. HDC identifies common patterns during learning and eliminates the saturation of the class hypervectors during single-pass training. Instead of naively combining all encoded data, our approach adds each encoded data to class hypervectors depending on how much new information the pattern adds to class hypervectors. If a data point already exists in a class hypervector, HDC will add no or a tiny portion of data to the model to prevent hypervector saturation. If the prediction matches the expected output, no update will be made to avoid overfitting. This adaptive update provides a higher chance and weight to non-common patterns to represent the final model. This method can eliminate the necessity of using costly iterative training.

Let us assume $\vec{\mathcal{H}}$ as a new training data point. HDC computes the cosine similarity of $\vec{\mathcal{H}}$ with all class hypervectors. We compute similarity of a data point with $\vec{\mathcal{C}_i}$ as: $\delta(\vec{\mathcal{H}}, \vec{\mathcal{C}_i})$. Instead of naively adding a data point to the model, HDC updates the model based on the δ similarity. If an input data has label l and correctly matches with the class, the model updates as follows:

$$\vec{\mathcal{C}_l} \leftarrow \vec{\mathcal{C}_l} + \eta_1 \ (1 - \delta_l) \times \vec{\mathcal{H}} \tag{S3}$$

where η is a learning rate. A large δ_l indicates that the input is a common data point which is already exist in the model. Therefore, our update adds a very small portion of encoded query to model to eliminate model saturation ($1 - \delta_l \simeq 0$). If the input data get an incorrect label of l', the model updates as:

$$\vec{\mathcal{C}_{l'}} \leftarrow \vec{\mathcal{C}_{l'}} - \eta_2 \ (\delta_{l'} - \delta_l) \times \vec{\mathcal{H}}$$

where $\delta_{l'} - \delta_l$ determines the weight that the model needs to be updated. Small $\delta_{l'} - \delta_l$ indicates that the query is marginally mismatched while larger mismatch is updated with a larger factor ($\delta_{l'} - \delta_l \gg 0$).

1.3 Hyperdimensional Inference

In inference, HDC checks the similarity of each encoded test data with the class hypervector in two steps. The first step encodes the input (the same encoding used for training) to produce a query hypervector $\vec{\mathcal{H}}$. Then we compute the similarity (δ) of $\vec{\mathcal{H}}$ and all class hypervectors. Query data gets the label of the class with the highest similarity.

2 Deep Continuous Local Learning (DECOLLE)

2.1 Neuron and Synapse Model

In DECOLLE, the neuron and synapse models follow leaky, current-based I&F dynamics with a relative refractory mechanism. The dynamics of the membrane potential U_i of a neuron i is governed by the following differential equations [97]:

$$\begin{aligned} U_i(t) &= V_i(t) - \rho R_i(t) + b_i, \\ \tau_{mem}\frac{\mathrm{d}}{\mathrm{dt}}V_i(t) &= -V_i(t) + I_i(t), \\ \tau_{ref}\frac{\mathrm{d}}{\mathrm{dt}}R_i(t) &= -R_i(t) + S_i(t), \end{aligned} \tag{S4}$$

with $S_i(t)$ the binary value representing whether neuron i spiked at time t. To facilitate implementation, the membrane potential is separated into two variables U and V. A spike is emitted when the membrane potential reaches a threshold $S_i(t) = \Theta(U_i(t))$, where $\Theta(x) = 0$ if $x < 0$, otherwise 1 is the unit step function. The constant b_i represents the intrinsic excitability of the neuron. The factors τ_{ref} and τ_{mem} are time constants of the membrane and refractory dynamics, respectively. I_i denotes the total synaptic current of neuron i, expressed as [97]:

$$\tau_{syn}\frac{d}{dt}I_i(t) = -I_i(t) + \sum_{j\in \text{ pre}} W_{ij}S_j(t), \tag{S5}$$

where W_{ij} is the synaptic weights between pre-synaptic neuron j and post-synaptic neuron i. Because V_i and I_i are linear with respect to the weights W_{ij}, The dynamics of V_i can be rewritten as [97]:

$$\begin{aligned} V_i(t) &= \sum_{j\in\text{pre}} W_{ij}P_{ij}(t), \\ \tau_{mem}\frac{\mathrm{d}}{\mathrm{dt}}P_{ij}(t) &= -P_{ij}(t) + Q_{ij}(t), \\ \tau_{syn}\frac{\mathrm{d}}{\mathrm{dt}}Q_{ij}(t) &= -Q_{ij}(t) + S_j(t). \end{aligned} \tag{S6}$$

The states P and Q describe the traces of the membrane and the current-based synapse, respectively. For each incoming spike, the trace Q undergoes a jump of height 1 and otherwise decays exponentially with a time constant τ_{syn} . Weighting the trace Q_{ij} with the synaptic weight W_{ij} results in the Post–Synaptic Potentials of neuron i caused by input neuron j. In the equation above, since P_{ij} and Q_{ij} are linear and are only driven by S_j, the index i can be dropped. This results in as many P and Q variables as there are presynaptic neurons, independently of the number of synapses.

Rewritten in discrete time with time step Δt and annotate the equation with the superscript l denoting the layer to which the neuron belongs, the dynamical equations above are:

$$\begin{aligned} U_i^l[t] &= \textstyle\sum_j W_{ij}^l P_j^l[t] - \rho R_i^l[t] + b_i^l, & P_j^l[t+\Delta t] &= \alpha P_j^l[t] + (1-\alpha)Q_j^l[t], \\ S_i^l[t] &= \Theta\left(U_i^l[t]\right), & Q_j^l[t+\Delta t] &= \beta Q_j^l[t] + (1-\beta)S_j^{l-1}[t], \\ & & R_i^l[t+\Delta t] &= \gamma R_i^l[t] + (1-\gamma)S_i^l[t], \end{aligned} \tag{S7}$$

where the constants $\alpha = \exp\left(-\frac{\Delta t}{\tau_{\text{mem}}}\right)$, $\gamma = \exp\left(-\frac{\Delta t}{\tau_{\text{ref}}}\right)$, and $\beta = \exp\left(-\frac{\Delta t}{\tau_{\text{syn}}}\right)$ reflect the decay dynamics of the membrane potential U, the refractory state R and the synaptic state Q during a Δt timestep.

2.2 DECOLLE Update Rule

As discussed above, in DECOLLE, we attach a random readout to each of the N layers of spiking neurons [97]:

$$Y_i^l = \sum_j G_{ij}^l S_j^l,$$

where G_{ij}^l are fixed, random matrices. The global loss function is then defined as the sum of the layerwise loss functions defined on the random readouts, i.e. $\mathcal{L} = \sum_{l=1}^N L^l\left(Y^l\right)$. To enforce locality, DECOLLE sets to zero all non-local gradients, i.e., $\frac{\partial L^l}{\partial W_{ij}^m} = 0$ if $m \neq l$. With this assumption, the weight updates at each layer become:

$$\Delta W_{ij}^l = -\eta \frac{\partial L^l}{\partial W_{ij}^l} = -\eta \frac{\partial L^l}{\partial S_i^l} \frac{\partial S_i^l}{\partial W_{ij}^l},$$

where η is the learning rate. Assuming the loss function depends only on variables in same time step, the first gradient term on the right hand side, $\frac{\partial L^l}{\partial S_i^l}$, can be trivially computed using the chain rule of derivatives. For the second gradient term, surrogate gradient-based learning gives [25]:

$$\frac{\partial S_i^l}{\partial W_{ij}^l} = \sigma'\left(U_i^l\right) \frac{\partial U_i^l}{\partial W_{ij}^l}$$

where $\sigma'\left(U_i^l\right)$ is the surrogate gradient of the non-differentiable step function $\Theta\left(U_i^l\right)$. In particular, a piecewise linear surrogate activation function is used, such that its derivative becomes the boxcar function $\sigma'(x) = 1$ if $x \in [-0.5, 0.5]$ and 0 otherwise. The rightmost term is computed as:

$$\frac{\partial U_i^l}{\partial W_{ij}^l} = P_j^l - \rho \frac{\partial R_i^l}{\partial W_{ij}^l}$$

Because regularization is used to favor low firing rates in DECOLLE, the terms involving R_i^l can be ignored. Combining the above equation give the DECOLLE rule governing the synaptic weight update:

$$\Delta W_{ij}^l = -\eta \frac{\partial L^l}{\partial S_i^l} \sigma'\left(U_i^l\right) P_j^l. \tag{S8}$$

3 SpikeHD Trainable parameters

Figure S2 demonstrates the effect of trainable parameter ratio on the performance of the model. To stress-test our model, the shape of the SNN is limited to 3 LIF layers with size ratio 2:3:2, and the dimension of HDC is derived from however many trainable parameter is allowed in HDC Memory, up to residue. For small SpikeHD (blue line with 10k parameters), there is a fine spot where optimal accuracy is reached. This is due to the fact that the training data requires an SNN big enough to extract meaningfull features (which explain the left slope) and an HDC memory large enough to memorize these features (which explain the right slope). Due to the fact that HDC memory accuracy resembles a sigmoid function with respect to dimension in practice, the accuracy deteriorate rapidly upon lowering the dimension to a certain threshold. This is demonstrated in the rapid increase in error rate for SpikeHD with very high SNN proportion. In addition, due to the correlation of the dimension threshold to the output dimension of the SNN instead of the size of the SNN, large SpikeHD (black line with 40k parameters) requires smaller proportion of HDC memory to perform well. The demand for the size of HDC also comes the number of classes. For example, the Omniglot dataset, a few-shot, 1623-class classification task, demands high HDC memory.

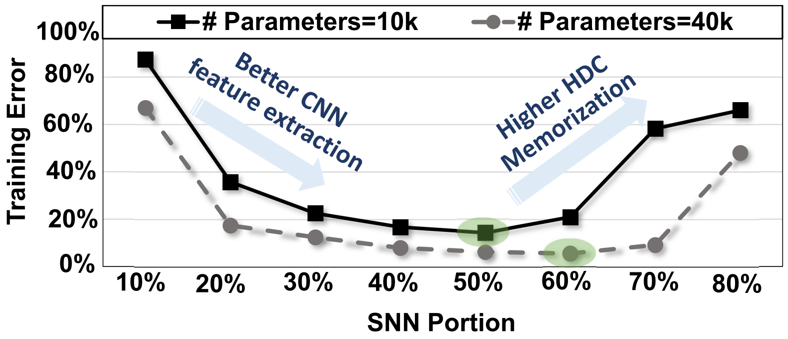

Figure S2: SpikeHD performance with different SNN/HDC training parameter ratios. The lines describe the average training accuracy of SpikeHD varying on the amount of trainable parameters that is assigned to SNN.

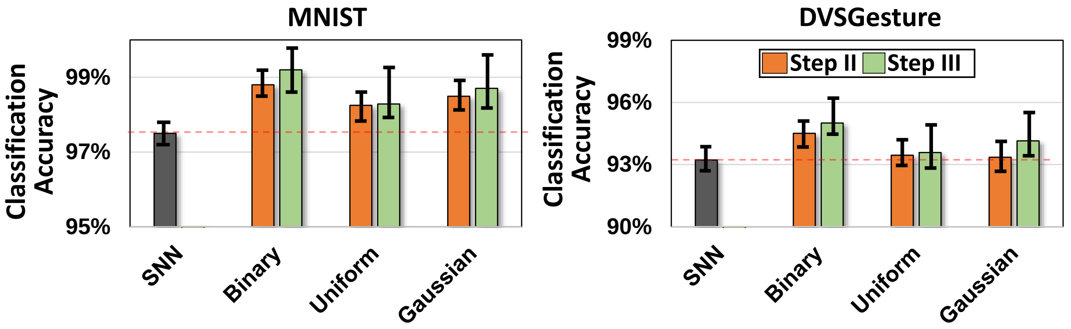

Figure S3: Training Performance of SpikeHD with convolutional SNN and distinct HDC nonlinear encoders on MNIST and DVSGesture. Each bar plot represents the accuracy of SNN along with SpikeHD using HDC encoders with binary, uniform, or Gaussian base hypervectors and with *Tanh* activation.

4 SpikeHD Enhancing Convolution-based SNN

We apply a convolutional architecture with two convolution layers of 16, 32 channels respectively and with kernel of size 7 and a fully connected layer of 64 neurons. Each convolutional LIF layers are followed by a max pool of kernel size 2. Figure S3 compares the test classification accuracy of SpikeHD with convolution-based SNN. For SNN, we use the conventional DECOLLE architecture in our default configuration. Our evaluation shows that default SpikeHD outperforms both SNN and HDC in terms of quality of learning. SpikeHD achieves, on average, 1.7% and 1.8% higher classification accuracy compared to the SNN model after co-training on MNIST and DVS Gesture, respectively.
